# Evidence for an early cadherin–catenin interaction network in ctenophores

**DOI:** 10.1093/molbev/msag123

**Published:** 2026-05-22

**Authors:** Lucas J Guttieres, Anhadvir Singh, Adriano Senatore, Mark Q Martindale

**Affiliations:** The Whitney Laboratory for Marine Bioscience, Department of Biology, University of Florida, Saint Augustine, FL, USA; Department of Biology, University of Toronto Mississauga, 3359 Mississauga Road, Mississauga, ON L5L 1C6, Canada; Department of Biology, University of Toronto Mississauga, 3359 Mississauga Road, Mississauga, ON L5L 1C6, Canada; The Whitney Laboratory for Marine Bioscience, Department of Biology, University of Florida, Saint Augustine, FL, USA

**Keywords:** cadherins, catenins, cell adhesion, ctenophores, evolution

## Abstract

The cadherin–catenin complex (CCC) is a calcium-dependent assembly that is essential for the organization and function of animal cells and tissues. CCC components form adherens junctions that link cell adhesion to the actin cytoskeleton and important signaling pathways that control processes, such as gene expression, cell polarity, and growth. While the CCC has been extensively studied and known to be conserved across most metazoan lineages, its occurrence in ctenophores, one of the earliest branching groups, has been questioned, with implications for the origins of multicellularity in animals. Here, we show that the ctenophore *Mnemiopsis leidyi* possesses a reduced cadherin repertoire yet retains conserved interactions characteristic of the CCC. Phylogenetic analyses identified a novel ctenophore-specific cadherin phylogenetically distant from major cadherin families from other animals. Screening a custom yeast two-hybrid library, derived from *M. leidyi* embryo cDNA, with the cytoplasmic tail of this noncanonical cadherin-like protein identified known CCC components β-catenin, p120, and Hakai as interacting proteins. Similarly, a screen using *M. leidyi* α-catenin as bait identified β-catenin, vinculin, and other known actin cytoskeleton-associated proteins. Directed yeast two-hybrid assays confirmed key interactions and demonstrated that targeted mutagenesis of conserved residues abolished binding, as is observed in other metazoans. Together, these findings suggest that core molecular interactions underlying the CCC are conserved in *M. leidyi*, consistent with the hypothesis that a functional CCC was an ancestral trait foundational to the evolution of multicellular animals.

## Introduction

The emergence of cell adhesion molecules (CAMs) that couple cell–cell adhesion to the cytoskeleton and intracellular signaling likely represented a critical step in the evolution of animal multicellularity. In metazoans, cell–cell and cell–substrate adhesions are largely mediated by CAMs, particularly cadherins and integrins (Halbleib and Nelson 2006; Iwamoto and Calderwood 2015). However, cadherins and integrins are absent from plants and fungi, which rely on alternative adhesion strategies (Kang et al. 2021; Atakhani et al. 2022). Nonetheless, organisms more phylogenetically proximal to animals do possess cadherins and integrins, such as Choanoflagellates, which possess a rich repertoire (Abedin and King 2008), and filastereans, such as *Capsaspora owczarzaki*, that harbor a single cadherin with an extensive array of integrin-related proteins (Sebé-Pedrós et al. 2013; Suga et al. 2013). Comparative genomic analyses of ichthyosporeans further indicate that integrin-related proteins were already present prior to the emergence of metazoans (Grau-Bové et al. 2017). Conversely, no cadherins have been detected in the social amoeba *Dictyostelium discoideum*, which has one copy of *α-catenin* and one of *β-catenin* in its genome. The interaction between these two proteins is essential for the formation of the fruiting body, a multicellular structure displaying epithelial-like organization (Dickinson et al. 2011). These observations suggest that components of the molecular toolkit required for cell–cell adhesion were already partially assembled in unicellular ancestors of metazoans.

**Figure 1:**
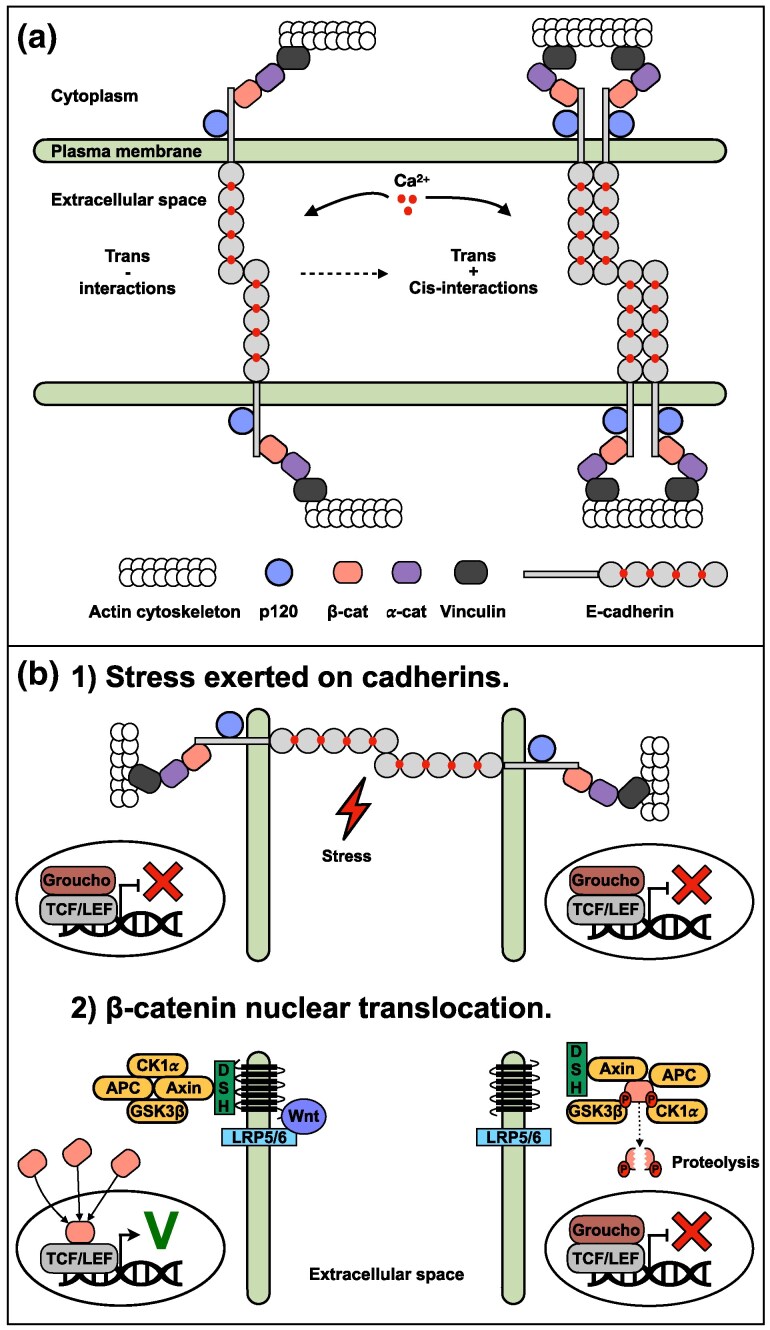
Extracellular and intracellular structure of AJs and signaling activity of the CCC. a) Schematic of AJ assembly. b) CCC-mediated β-catenin signaling: (1) mechanical stress or other perturbations can disrupt junctional complexes; (2) degradation of cadherin interactions releases β-catenin, enabling its nuclear translocation and transcriptional activation through TCF/LEF. APC, adenomatous polyposis coli; CK1*α*, casein kinase 1*α*; DSH, disheveled; GSK3β, glycogen synthase kinase 3β; LEF, lymphoid enhancer factor; LRP, low-density lipoprotein receptor-related protein; TCF, T-cell factor; Vin, vinculin; *α*-cat, *α*-catenin; β-cat, β-catenin.

The emergence of multicellularity is closely associated with the emergence of a novel mode of tissue organization: the epithelium (Leys and Riesgo 2012). Epithelial tissues form polarized cell layers that act as physical barriers and provide structural integrity, functions that critically depend on robust intercellular adhesion (Fu et al. 2022). In the epithelium, the cadherin–catenin complex (CCC) forms mechanosensitive structures called adherens junctions (AJs), which couple classical cadherins at the plasma membrane to the actin cytoskeleton through interactions with cytoplasmic catenins (Aberle et al. 1996b; Huber et al. 2001; Yamada et al. 2005). The cytoplasmic tail of classical cadherins binds β-catenin, which in turn recruits α-catenin, thereby connecting the complex to the actin cytoskeleton. Adhesion between neighboring cells is mediated by the extracellular cadherin (EC) repeat domains, which contain calcium-binding sites and engage in homophilic trans interactions with cadherins on adjacent cells, adopting a rigid conformation (Fig. 1a; Nagar et al. 1996). These initial trans interactions are subsequently reinforced by lateral cis interactions, leading to clustering of cadherins and strengthening of cell–cell adhesion.

Beyond its structural role in maintaining AJs, the CCC is also involved in multiple intracellular signaling pathways, most notably by regulating the cytoplasmic availability of β-catenin through the release from the cadherins at the membrane. Cadherins are subjected to multiple forms of stress, including mechanical stress (eg AJ remodeling and cell division) as well as enzymatic stress mediated by kinases, which can induce cadherin phosphorylation followed by degradation (Fig. 1b; Canel et al. 2013; Aguilar-Aragon et al. 2020). Upon cadherin degradation, β-catenin is no longer sequestered by the cytoplasmic tail of classical cadherins and thus becomes available in the cytoplasm, where its fate is determined by the canonical Wnt signaling pathway (cWNT; Stamos and Weis 2013). When β-catenin is released into the cytoplasm under active cWnt signaling, the β-catenin destruction complex is inhibited, allowing β-catenin to translocate to the nucleus and activate transcriptional programs (eg germ layer specification in vertebrates; Farge 2003; Gayrard et al. 2018; Röper et al. 2018).

Within animals, recent studies have extended the conservation of CCC functions to nonbilaterian lineages. In the cnidarian *Nematostella vectensis*, the CCC components ubiquitously co-localize and are essential for cell–cell adhesion, embryogenesis, and germ layer formation (Clarke et al. 2016, 2019; Pukhlyakova et al. 2019). In placozoans, the genomic presence of CCC components together with evidence for calcium-dependent AJs in *Trichoplax adhaerens* supports the conservation of this complex in this lineage, although functional studies remain absent (Hulpiau and van Roy 2011; Smith and Reese 2016). In sponges, all CCC components have been shown to co-localize at cell–cell boundaries, and their physical interactions have been demonstrated by co-immunoprecipitation in the freshwater sponge *Ephydatia muelleri* and by yeast two-hybrid (Y2H) screening in the marine sponge *Oscarella carmela* (Nichols et al. 2012; Schippers and Nichols 2018).

Despite the important role of the CCC in metazoan development, no investigations have been conducted in one of the earliest-branching animal lineage, the ctenophores (Dunn et al. 2008; Hejnol et al. 2009; Whelan et al. 2017; Schultz et al. 2023). A previous comparative genomic study raised doubts about the conservation of a canonical CCC in *Mnemiopsis leidyi*, reporting that one of its cadherins lacks key catenin-binding motifs (Belahbib et al. 2018). However, immunohistochemistry using custom *Ml*-βcatenin polyclonal antibodies recently revealed strong β-catenin enrichment at cell–cell contacts during *M. leidyi* embryogenesis and therefore suggested a potential conserved role in cell adhesion (Walters et al. 2025). Here, we analyzed the full repertoire of CCC homologs encoded by three ctenophore species and found that most critical residues and protein domains mediating these interactions are highly conserved. Using the Y2H screening system, we identified canonical interactions among core CCC components in *M. leidyi*. Furthermore, targeted mutation of key residues and domains involved in the interactions between β-catenin, α-catenin, and the nonclassical cadherin-like identified in this study completely abolished protein binding between the canonical CCC members, supporting the putative existence of a functional CCC in ctenophores.

## Results

### Identification and phylogenetic characterization of the catenin proteins in ctenophores

**Figure 2:**
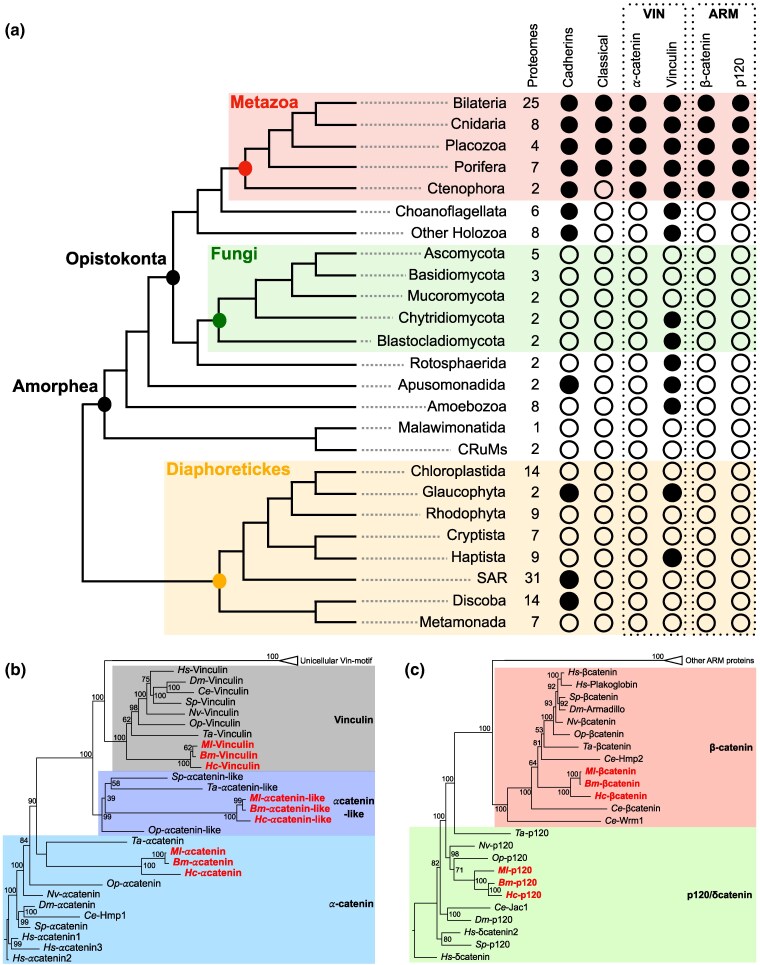
Evolutionary distribution of the CCC components across the eukaryotic kingdom. a) Presence/absence of cadherins, Vinculin-family, and Armadillo-family proteins across major eukaryotic lineages, screened using HMM-based searches. Filled and open circles indicate presence or absence, respectively. b) Maximum-likelihood phylogenetic tree of Vin-family proteins from eukaryotes. c) Maximum-likelihood phylogenetic tree of ARM-domains focusing on β-catenin and p120. The full trees are depicted in Figs. S1 and S2. Nodes for both trees are supported by approximate Bayes tests. Ctenophore proteins are highlighted in red. Species abbreviations: *Bm*, *Bolinopsis microptera*; *Ce*, *Caenorhabditis elegans*; *Dm*, *Drosophila melanogaster*; *Hc*, *Hormiphora californiensis*; *Hs*, *Homo sapiens*; *Ml*, *Mnemiopsis leidyi*; *Nv*, *Nematostella vectensis*; *Op*, *Oscarella pearsei*; *Sp*, *Strongylocentrotus purpuratus*; *Ta*, *Trichoplax adhaerens*.

To date, cadherins have been reported as exclusive to holozoans, being reported as absent in plants and fungi (Miller et al. 2013). To expand on previous analyses, we trained profile hidden Markov models (HMMs) using a set of bona fide metazoan cadherin homologs and used this model to search for homologs within an expanded set of eukaryotic proteomes (Fig. 2a; Senatore et al. 2025). This approach identified putative cadherin homologs in Apusomonadida and three major lineages within Diaphoreticka (Glaucophyta, SAR, and Discoba), suggesting they originated earlier in eukaryotic evolution than previously thought (Fig. 2a). In contrast, we did not detect cadherin proteins in plants or fungi, consistent with previous studies.

Searching for Vinculin-motif–containing proteins (Vin-motif), including α-catenin, α-catulin, and vinculin, yielded 219 nonredundant sequences, including three Vin-superfamily homologs from *M. leidyi* and *Hormiphora californiensis* (Data S5). Additionally, we also found three homologs in *Bolinopsis microptera* in the NCBI database. Next, we performed a phylogenetic analysis with the ctenophore homologs and representative α-catenin and vinculin sequences from other metazoans and unicellular eukaryotes (Fig. 2b). This analysis revealed that ctenophore homologs segregate into three distinct groups. One group clusters with canonical nonbilaterian α-catenins, in a clade distinct from bilaterian and cnidarian α-catenins, whereas a second group falls within a previously described uncharacterized α-catenin-like clade (Miller et al. 2013). The third group clusters with vinculin proteins, which are the closest relatives of Vin-motif homologs from unicellular eukaryotes. In contrast, no α-catulin homologs were detected in ctenophores nor other nonbilaterian lineages except for cnidarians, nor in unicellular relatives of metazoans.

Next, we searched for β-catenin and related proteins. Since these comprised armadillo (ARM) repeat domains, which tend to have low overall sequence similarity, we trained HMMs exclusively on β-catenin and p120 sequences in order to restrict the search space (Gul et al. 2017). This analysis yielded 758 nonredundant proteins, including six from *M. leidyi* and *H. californiensis* (Data S6). The β-catenin and p120 sequences from *B. microptera* were retrieved from the NCBI database. A phylogenetic analysis of representative homologs, with sequences from major taxonomic groups, identified two candidates in each ctenophore species with strongly supported clade relationships with β-catenin and p120/δ-catenin homologs from other animals (Fig. 2c). *M. leidyi* β-catenin was previously identified in another study, shown to have high sequence conservation with other metazoan β-catenin homologs (Walters et al. 2025). *M. leidyi* β-catenin contains 11 out of the 12 canonical ARM repeat domains, with repeat 7 predicted with low confidence due to the apparent absence of a third α-helix. However, structural analyses of human β-catenin have revealed that ARM repeat 7 naturally consists of only two α-helices, rather than the three typically observed in other ARM repeats (Xing et al. 2008). Thus, the low-confidence prediction of ARM repeat 7 in the *M. leidyi* homolog might reflect this atypical structural feature, supporting the presence of a complete set of 12 ARM repeats. In addition, we identified a single p120 homolog, a key positive regulator of the CCC that prevents the endocytosis of classical cadherins (Davis et al. 2003). Notably, no p120 orthologs were detected outside of metazoans, suggesting that p120 may represent a metazoan-specific innovation (Fig. 2a).

### Identification of a putative noncanonical cadherin-like in ctenophores

**Figure 3:**
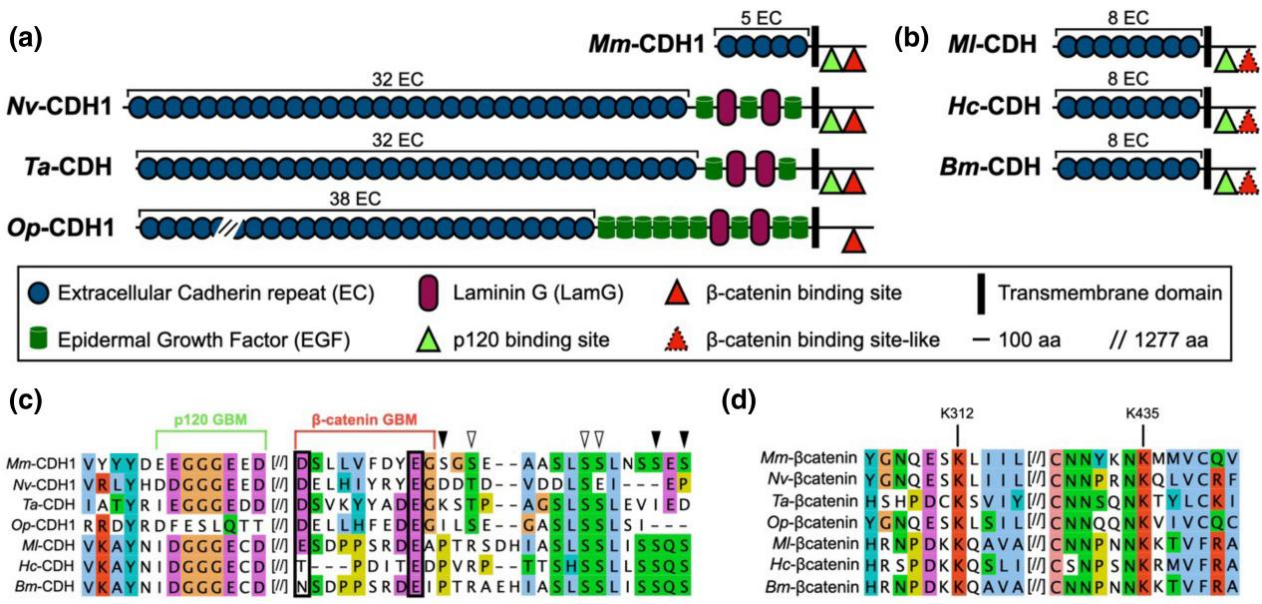
Conservation of the CCC in ctenophores. a) Representative classical cadherins from a vertebrate (*Mus musculus*, *Mm*) and from nonbilaterian phyla, including Cnidaria (*Nematostella vectensis*, *Nv*), Placozoa (*Trichoplax adhaerens*, *Ta*), and Porifera (*Oscarella pearsei*, *Op*). b) Predicted domain architecture of the ctenophore nonclassical cadherin-like repertoire identified. Dotted triangle indicates imperfect conservation. c) Alignment of the p120- and β-catenin–binding GBMs. Critical residues required for β-catenin binding are highlighted with black boxes. Phosphorylation sites for CKII and GSK3β are indicated by upside down filled or open triangles, respectively. The full alignment of intracellular domains of selected classical cadherins is provided in Fig. S5. d) Alignment of the two lysines K312 and K435 involved in classical cadherin binding from selected metazoan β-catenin. A full alignment of the β-catenin sequences is available in Fig. S6.

To explore whether adhesion-related functions of classical cadherins might extend to ctenophores, we investigated the conservation of known cadherin/β-catenin interaction sequences and structures. Classical cadherins are characterized by a conserved intracellular catenin-binding region for p120- and β-catenin, whereas their extracellular architecture diversified during animal evolution. In contrast to vertebrate classical cadherins, those of invertebrate lineages typically contain epidermal growth factor (EGF) and laminin G (LamG) domains (Fig. 3a; Oda and Takeichi 2011). Nevertheless, the absence of these domains does not preclude classification as a classical cadherin, as they exhibit lineage-specific gain and loss across metazoans.

From our HMM search, we identified a total of six cadherin gene candidates in *M. leidyi* and five in *H. californiensis* and *B. microptera* (Fig. S3). Domain annotation using Pfam did not yield a clear classification of these proteins into known cadherin subfamilies (Hulpiau and van Roy 2011). We therefore performed a motif-based analysis using MEME to search for conserved sequence features beyond canonical EC repeats. From this analysis, we identified one cadherin that displayed one conserved p120-binding site but lacked a clear β-catenin binding site in each ctenophore species (Fig. 3b). Additionally, we found a prospective protocadherin (PCDH) containing a CM1-like cytoplasmic motif, characteristic of nonclustered PCDHs in *M. leidyi* and *H. californiensis* (Fig. S4; Hulpiau and van Roy 2011). Consistent with previous studies, *Ml*-PCDH and *Hc*-PCDH lack the CM2 motif, which is absent in some protostomes and nonbilaterian lineages (Hulpiau and van Roy 2011). Surprisingly, we did not detect any extremely long cadherins characteristic of the Fat, Fat-like, or Dachsous families, nor any containing the characteristic seven transmembrane domains of CELSR/Flamingo. This absence suggests that these major cadherin families likely emerged later in metazoans.

To determine whether any of the ctenophore cadherins contain sequence elements critical for CCC assembly in other animals, we performed independent alignments with classical cadherins from selected metazoan groups. Remarkably, one cadherin candidate in each of the three ctenophore species (*Ml*-CDH, *Hc*-CDH, and *Bm*-CDH) share a common architecture: eight EC repeats, one transmembrane domain, and a modified classical cadherin cytoplasmic domain. Indeed, they contain a well-conserved p120-catenin groove binding motif (GBM) (X-X-[ED]-G-G-G-E), including the critical three glycine residues required for cadherin–p120 interaction (Fig. 3b and c; Ishiyama et al. 2010). Additionally, the residues of *Ml*-p120 involved in interaction with the cytoplasmic tail of classical cadherins are highly conserved (Belahbib et al. 2018). By contrast, the β-catenin GBM (D–X–X–X–X–aromatic–X–X–E–G) is only partially conserved in the ctenophore cadherins: The aspartate is replaced by another acidic residue in *Ml*-CDH (glutamate) or a nonconserved amino acid substitution in *Hc*-CDH and *Bm*-CDH; the aromatic and the glycine residues are not conserved. However, the aspartate at the end of the β-catenin GBM is highly conserved in the three ctenophore cadherins (Fig. 3c). Given this amino acid composition, we describe the region as β-catenin binding site-like rather than a canonical β-catenin binding site. Additionally, four out of six serine residues corresponding to previously described GSK3β and casein kinase 2 (CK2) phosphorylation sites involved in the regulation of cadherin-mediated adhesion dynamics are conserved in *Ml*-CDH, *Hc*-CDH, and *Bm*-CDH (Fig. 3c; Gooding et al. 2004).

**Figure 4:**
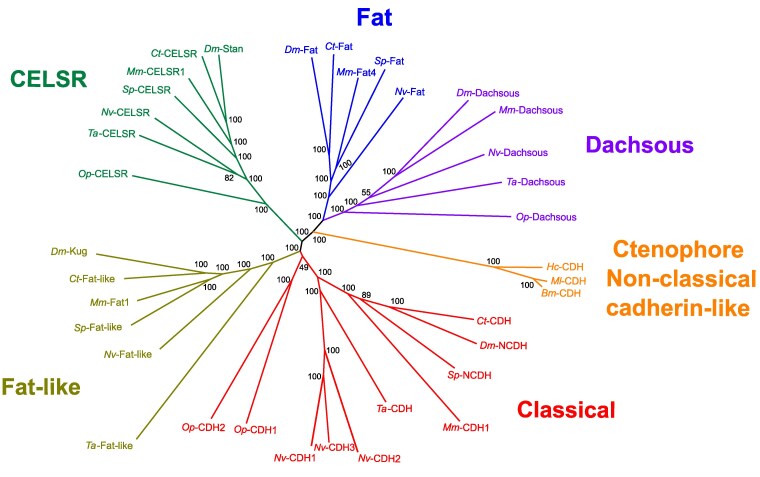
Phylogenetic relationships of cadherin superfamily members across metazoans. Maximum-likelihood tree showing representative members of major cadherin families, including classical, Fat, Fat-like, Dachsous, and CELSR/Flamingo. Selected sequences from bilaterians, cnidarians, placozoans, sponges, and ctenophores are color coded by family. Nodal support values reflect approximate Bayes test values at key nodes. Ctenophore cadherins does not cluster within the classical cadherin clade but instead branches separately, highlighting their status as non bona fide classical cadherins. Species abbreviations are as Fig. 2 in which we added *Ct*, *Capitella teleta*.

The presence of p120- and β-catenin–binding sites is not restricted to classical cadherins. Indeed, the cytoplasmic domains of Dachsous cadherins from other animals also contain similar binding motifs, although no direct interaction with β-catenin has been clearly demonstrated (Clark et al. 1995; Clarke et al. 2016). To determine the phylogenetic relationship of ctenophore cadherins relative to major cadherin families, we performed a phylogenetic analysis using a modified dataset of previously annotated cadherins (Hulpiau and van Roy 2011). Maximum-likelihood analyses resolved the major cadherin families into well-supported clades, including classical cadherins, Fat, Fat-like, Dachsous, and CELSR/Flamingo (Fig. 4). These cadherin families formed distinct and robustly supported clades. Surprisingly, ctenophore cadherins did not group with either classical or Dachsous cadherins but instead occupied a distinct phylogenetic position outside all major cadherin families. Although alternative rooting strategies influence the inferred order of early divergences within the cadherin superfamily, both the clustering of the major cadherin families and the placement of ctenophore cadherins outside the classical cadherin clade are robust. Based on this phylogenetic position and the composition of the β-catenin–binding motif, we refer to these proteins as nonclassical cadherin-like rather than true classical.

We also examined the β-catenin protein sequences from the three ctenophore species to assess the possible conservation of the critical residues mediating interactions with a classical cadherin and *α*-catenin. The two critical lysines K312 and K435 that interact with D674 and E682 of the β-catenin GBM in classical cadherins are conserved (Fig. 3d; Huber et al. 2001). Furthermore, most residues involved in the interaction with α-catenin are also conserved, with minor substitutions (Fig. S6). Reciprocally, the hydrophobic residues mediating the interaction between the two proteins are mostly conserved in ctenophore α-catenin proteins (Fig. S7). Together, these data suggest that ctenophores may possess a modified form of the CCC.

### The CCC components interact in vitro in *M. leidyi*

Our identification of a nonclassical cadherin-like in *M. leidyi*, along with homologs for the core components of the CCC, spurred us to determine experimentally whether these proteins can physically interact with each other. Previous studies reported that the Y2H assay was an efficient strategy for the detection of protein–protein interaction between the CCC components (Nieset et al. 1997; Simcha et al. 2001; Nichols et al. 2012). Thus, we generated a custom Y2H cDNA library derived from one-cell to 8 hpf *M. leidyi* embryos, which we could use to screen proteins of interest. We initially attempted to use *Ml*β-catenin as the bait to screen the library, but unfortunately, the full-length coding sequence resulted in autoactivation of reporter genes. As an alternative approach, we conducted two separate Y2H screens using the intracellular domain of *Ml*-CDH and *Ml*-αcatenin as baits. These screens identified a total of seven putative interacting proteins for *Ml*-CDH and 13 for α-catenin (Table S1). To validate these interactions, we performed directed Y2H assays using the minimal interacting domains identified from the initial screenings (Table S3). From this second set of experiments, we confirmed three interactions for *Ml*-CDH and six for *Ml*-αcatenin (Table 1). Notably, both screenings recovered β-catenin as a binding partner, consistent with conserved interactions within the CCC in *M. leidyi*. Our data also revealed interactions with two major regulators of E-cadherins. First, we identified p120 as a binding partner, consistent with the presence of a well-conserved p120-binding site in the cytoplasmic tail of *Ml*-CDH (Fig. 3c). Secondly, we detected an interaction with Hakai, which promotes ubiquitination of phosphorylated E-cadherins in vertebrates by recognizing phosphorylated Y755 in mouse E-cadherin upstream of the p120-binding motif via its Hakai phosphotyrosine-binding (HYB) domain (Fujita et al. 2002; Mukherjee et al. 2012). Interestingly, this tyrosine is substituted in nonbilaterian and tissue-specific classical cadherins, except vertebrate E-cadherins (Fig. 3c). Conversely, *Ml*-Hakai contains all the key residues required for E-cadherin binding, including the zinc-coordinating residues that form the HYB domain (Fig. S8).

**Table 1:** Binding partners of the *Ml*-CDH intracellular domain and *Ml*-α-catenin identified by Y2H screening and validated by directed Y2H assays.

Gene ID	Gene identification	Major predicted domains
**Intracellular domain of *Ml*-CDH**		
ML073715a	β-Catenin	12 × Armadillo (ARM)
ML009118a	Hakai	Zinc finger RING-type; zinc finger C2H2
ML002622a	p120	6 × ARM
***Ml*-** *α* **catenin**		
ML073715a	β-Catenin	12 × ARM
ML148910a	Vinculin	4 × VIN
ML084414a	Merlin	Four-point-one, Ezrin, Radixin, and Moesin domain (FERM)
ML31032a	Afadin	2 × Ras-associating (RA); forkhead-associated (FHA)
ML02953a	Diaphanous	Diaphanous GTPase binding domain; Formin FH3; Formin FH2
ML25062a	*α*-Actinin	2 × calponin homology (CH); 3 × spectrin repeat; 2 × EF-hand

Protein domains were predicted using InterPro and SMART and are listed from the N-terminus to the C-terminus. The complete list of predicted binding interactions from preliminary screenings is provided in Table S2.

At AJs, α-catenin binds β-catenin associated with the cytoplasmic tail of cadherins at the plasma membrane and connects this complex to the actin cytoskeleton by interacting with F-actin and multiple actin proteins. Our screening performed with *Ml*-αcatenin predicted interaction with five actin proteins that are known, in vertebrates, to physically interact with α-catenin, suggesting a conserved mechanism in linking the actin cytoskeleton to the membrane in *M. leidyi* (Takeichi and Abe 2005).

### Mutation of critical residues abolishes interaction between CCC members

**Figure 5:**
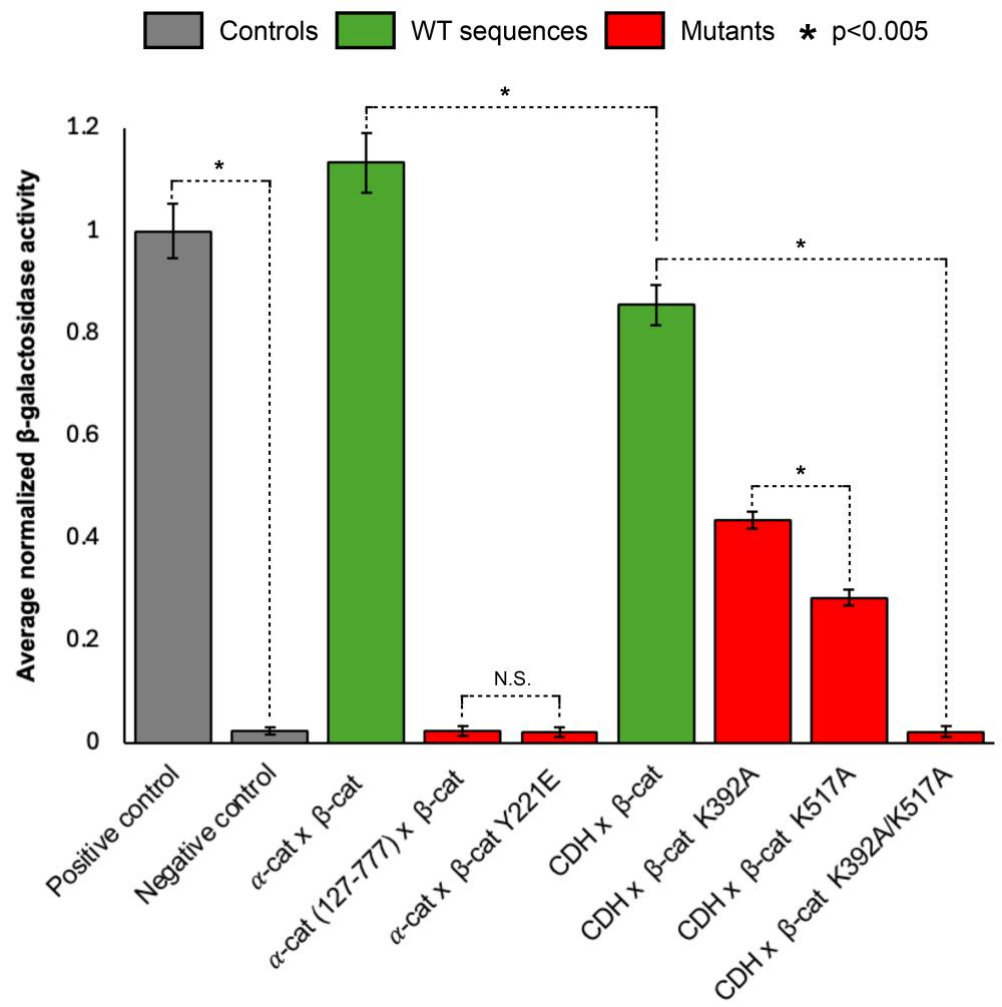
Mutation of critical residue block interactions between the canonical CCC components. The β-galactosidase activity was measured and normalized to a known positive interaction (mouse p53 and SV40 large T antigen). Dashed lines and asterisks indicate statistically significant differences as determined by two-sample *t*-tests.

The core CCC forms a stable scaffold at AJs, while its assembly and maintenance is dynamically regulated, all mediated by critical residues that have been well characterized (Aberle et al. 1996a, 1996b; Huber et al. 2001). Remarkably, the most essential residues are highly conserved in *M. leidyi* (Fig. 3c and d; Figs. S6 and S7). To further assess the interactions between β-catenin, α-catenin, and the nonclassical cadherin-like identified from the Y2H screens and directed yeast experiments, we tested the impact of single amino acid mutations and domain truncations by quantifying the interaction strength through measurement of the β-galactosidase activity (Fig. 5). We observed that the interaction between *Ml*-β-catenin and *Ml*-α-catenin was significantly stronger than with the cytoplasmic tail of *Ml*-CDH. This difference may reflect the larger interaction interface with α-catenin compared to the β-catenin GBM in classical cadherins, which consists of only ten amino acids.

The interaction between β-catenin and α-catenin is tightly regulated by phosphorylation of Y142 in β-catenin and by the presence of a highly hydrophobic domain in the N-terminus part of α-catenin (Fig. S7; Huber et al. 1997; Piedra et al. 2003). In our study, a phosphomimetic mutation of the corresponding residue Y221E in *Ml*-β-catenin abolished α-catenin binding, suggesting a similar form of regulation could exist in ctenophores. This interaction is disrupted by the activity of the Src-kinase Fer and promotes β-catenin nuclear translocation (Náger et al. 2015). A quick reciprocal blast search on NCBI revealed that one Fer-candidate is present in the *M. leidyi* genome (GenBank: KAL5266948.1), suggesting a conserved phosphorylation-dependent mechanism in ctenophores. Separately, we also truncated the hydrophobic N-terminal β-catenin–binding domain of *Ml*-α-catenin, which yielded a similar loss of interaction.

The interaction between β-catenin and a classical cadherin mostly relies on two lysines (K312 and K435) that physically interact with D674 and E682, as exemplified by mouse classical cadherins (Fig. 3d; Huber et al. 2001). To further study the interaction between *Ml*-β-catenin and *Ml*-CDH, we mutated K392 and K517 to alanine to specifically remove their positive charges while minimally perturbing the overall structure of *Ml*-β-catenin. Mutation of either lysine partially reduced *Ml*-CDH binding, whereas simultaneous mutation of both residues completely abolished the interaction (Fig. 5). These results indicate that both lysines contribute synergistically to the electrostatic interface between β-catenin and the cytoplasmic tail of classical cadherins. Our data also show a differential impact of lysine mutations on the interaction. Mutation of K392A resulted in a milder reduction of binding than mutation of K517A. This observation suggests that the first lysine may be less critical for the interaction, which could explain why substitution of an aspartate by a glutamate within the β-catenin binding site–like motif of *Ml*-CDH did not prevent interaction with *Ml*-β-catenin (Fig. 3c).

## Discussion

### Evidence for conserved interactions within the CCC in *M. leidyi*

The CCC represents an ancestral molecular assembly that is deeply conserved across metazoans and may have played a key role in the transition from unicellularity to multicellularity (Nichols et al. 2012; Miller et al. 2013; Clarke et al. 2016). Conservation of the CCC has been documented from sponges to mammals, highlighting its fundamental role in cell–cell adhesion and tissue organization. However, its presence and composition in ctenophores remained unclear.

A previous study questioned the presence of the CCC in *M. leidyi* based on the apparent absence of catenin-binding sites in a single cadherin gene (Belahbib et al. 2018). By examining all cadherin candidates in three ctenophore species, our results refine this conclusion and suggest that a noncanonical CCC-like interaction network may exist in ctenophores, potentially mediated by a nonclassical cadherin-like protein. Although these nonclassical cadherin-like sequences show differences in the β-catenin–binding region, compared to other metazoan lineages, these substitutions are largely conservative and unlikely to abolish complex formation. Structural studies indicate that the cadherin–β-catenin interface relies on multiple cooperative contacts rather than single invariant residues allowing for some sequence plasticity (Huber et al. 1997, 2001). Similarly, ctenophore β-catenin proteins retain the two critical lysines that interact with the two acidic residues from the β-catenin GBM. However, in the three nonclassical cadherin-like proteins identified in ctenophores, only the terminal aspartate of the GBM is conserved, whereas the glutamate residue is not. Consistent with this observation, our data indicate that the second lysine plays a more prominent role than the first in mediating the β-catenin–cadherin interaction. Together, these results suggest that although the β-catenin–cadherin interaction may be retained in ctenophores, it may rely on a modified interaction interface, potentially resulting in a weaker or mechanistically distinct mode of binding compared to other metazoan lineages. Comparative data from another ctenophore species, *Bolinopsis mikado*, suggest that partial loss of nonessential residues does not prevent binding to E-cadherin in vitro (Mbogo et al. 2024). Together with the retention of most ARM repeats and the α-catenin–binding region, these observations indicate that the molecular interfaces required for CCC assembly are largely conserved in ctenophores despite domain loss and sequence modification. Importantly, our Y2H experiments combined with targeted mutagenesis approaches provide evidence that these components can interact in vitro. The conservation of these interactions could suggest that the CCC may tolerate sequence variation while preserving adhesive function, a feature that could have facilitated early multicellular organization. Using single-cell data previously produced in a *M. leidyi* adult lobate, we observed co-expression of the three CCC components in some single-cell clusters, notably in epithelial, neuronal, and muscle cell types (Fig. S9; Sebé-Pedrós et al. 2018). This observation combined with the localization of *Ml*-β-catenin at the plasma membrane during embryogenesis suggests that these interactions are physiologically relevant and that the CCC might be functionally assembled and promote cell adhesion in vivo in ctenophores (Walters et al. 2025). These results are consistent with the idea that the core CCC predates the emergence of morphologically complex tissues and was already present in early-branching metazoans. In turn, cadherin-mediated adhesion may have been a foundational feature of early metazoan multicellularity, evolving prior to the diversification of canonical cadherin architectures in later metazoans. While our data are consistent with the conservation of interaction interfaces within the CCC, alternative evolutionary scenarios should be considered. That is, we cannot exclude that these components represent a more modular or partial “adhesion toolkit” in ctenophores, rather than a fully integrated complex, which would have been subsequently incorporated into canonical AJs in other metazoan lineages.

### Regulation of cell adhesion dynamics in *M. leidyi*

Cell–cell adhesion mediated by the CCC is dynamic and relies on a balance between junction stabilization and turnover (Davis et al. 2003; Baum et al. 2011). This balance is controlled by coordinated interactions between cadherins and catenins, regulatory binding partners, and posttranslational modifications. Assembly of the core CCC provides basal adhesion, whereas p120 and β-catenin binding to the juxtamembrane domain acts as a key determinant of cadherin retention at the plasma membrane (Huber et al. 1997, 2001; Roura et al. 1999). The phosphorylation of cadherins by CK2 and GSK3β has been shown to regulate cadherin stability in a context-dependent manner, promoting membrane retention when p120 is bound, but facilitating turnover in its absence (McEwen et al. 2014). The conservation of several CK2 and GSK3β phosphorylation sites in *Ml*-CDH therefore suggests that similar regulatory principles may operate in ctenophores. While we did not detect interaction between *Ml*-CDH and these two kinases using the Y2H system, this likely reflects methodological limitations as kinase–substrate interactions are often transient and difficult to capture in Y2H assays (Ito et al. 2001; Linding et al. 2007). Therefore, we cannot exclude or confirm a conserved phosphorylation-dependent regulatory mechanism in ctenophores.

Conversely, our Y2H data predict interactions between the cytoplasmic tail of *Ml*-CDH and both p120 and Hakai. In vertebrate epithelial cells, these two proteins bind to overlapping regions within the juxtamembrane domain of E-cadherins and exert opposing effects on cadherin stability, with p120-catenin stabilizing cadherins at the plasma membrane and Hakai promoting their ubiquitination and endocytosis (Piedra et al. 2003; Ishiyama et al. 2010). In vertebrates, Src-dependent phosphorylation of tyrosines Y753 and Y754 in the E-cadherin cytoplasmic domain promotes recruitment of Hakai via its HYB domain, leading to cadherin ubiquitination, and AJ disassembly, a process that can facilitate β-catenin nuclear translocation (Qi et al. 2006;Mukherjee et al. 2012; Gayrard et al. 2018). While these two tyrosines are absent from invertebrate classical cadherins, an interaction with Hakai is nonetheless still possible. For instance, *Drosophila melanogaster* Shotgun lacks these residues but remains capable of interacting with Hakai, whereby the Hakai–cadherin interaction may not depend on a phosphotyrosine-based recognition mechanism, raising the possibility that this regulatory mechanism is vertebrate specific (Kaido et al. 2009). This possibility is further supported by the ability of *Ml*-CDH to interact with Hakai in vitro, together with the full conservation of residues implicated in E-cadherin recognition. Thus, our results suggest that key elements of the molecular framework underlying regulated cadherin turnover were already present prior to the diversification of E-cadherins and the emergence of specialized junctional architectures, such as AJs.

In extant epithelia, AJs respond dynamically to changes in tension via the linkage between the CCC and the actin cytoskeleton (Lecuit and Yap 2015). This connection is largely mediated by α-catenin, which can engage multiple actin-binding proteins (Takeichi and Abe 2005). Our data predict that several of these cytoskeletal regulators may interact with *Ml*-α-catenin (Table 1). Such interactions are often context dependent or require additional factors, such as actin itself, which can limit their detectability in a Y2H assay. Nonetheless, previous studies have shown that α-catenin can still interact with certain actin partners despite the divergence of the actin cytoskeleton in yeast, consistent with our observations (Nieset et al. 1997; Kobielak et al. 2004). While the limitations of Y2H prevent us from excluding additional interactions in *M. leidyi*, this approach provides initial insights into the regulation of the CCC and suggests a putative conserved function in cell adhesion. Given the current limitations of the experimental toolkit in ctenophores, many approaches commonly used in bilaterian model systems to assess in vivo gene functions remain challenging to implement. Further investigations will be needed to assess the physiological relevance of these identified CCC protein–protein interactions, for example, through protein localization and the development and use of gene perturbation approaches in the context of adhesion dynamics.

### Building multicellularity with a limited cadherin toolkit

**Figure 6:**
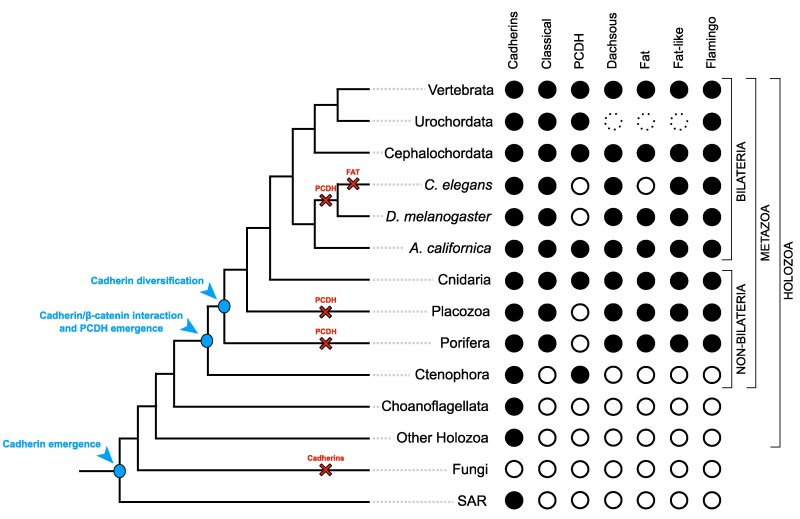
Proposed evolutionary history of major cadherin families. Columns correspond to major cadherin subfamilies. Filled and open circles indicate the presence or absence of major cadherin subfamilies, respectively, while dotted circles denote a potential presence in Urochordata (see main text for details). Chevrons and associated circles indicate putative evolutionary events. Cadherin diversification includes the inferred emergence of classical, Dachsous, Fat, Fat-like, and Flamingo cadherin families. Loss of specific cadherin subfamilies is indicated by “X” symbols, with the corresponding family labeled above.

The rich cadherin repertoire in choanoflagellates raises intriguing questions about their role in the transition to multicellularity (Abedin and King 2008; Rokas 2008). However, none of them are known to bind β-catenin, and their putative function in cell adhesion remains unclear. In metazoans, cadherins have diversified into multiple families, each with distinct domain architectures and functional specializations that are mostly conserved in all animals (Fig. 6; Tepass et al. 2000; Hulpiau and van Roy 2011; Niessen et al. 2011). No clear orthologs of Fat, Fat-like, or Dachsous could be confidently identified in *Ciona intestinalis*, possibly due to limitations in assembling large cadherin genes (Noda and Satoh 2008). Fat, Fat-like, Dachsous, and CELSR/Flamingo are known to play functions in signaling in Hippo and the WNT/planar cell polarity (Wnt/PCP) (Willecke et al. 2006; Goodrich and Strutt 2011; Butler and Wallingford 2017). Our data indicate that these cadherin families are absent in ctenophores, supporting the hypothesis that core cadherin components of the Hippo and WNT/PCP pathways arose after the divergence of ctenophores. This conclusion is consistent with genomic analyses showing that most essential components of both pathways are absent or highly divergent in ctenophores (Sebé-Pedrós et al. 2012; Ryan et al. 2013). Our study shows that despite this reduced repertoire, *M. leidyi* possesses a nonclassical cadherin-like protein capable of binding β-catenin. Although β-catenin binding is a hallmark of classical cadherins, this interaction is not exclusive to this family, as several cadherins have been reported to associate with β-catenin via serine-rich regions in their cytoplasmic tails instead of a canonical β-catenin GBM (Chen et al. 2002; de Nys et al. 2024). Taken together, these observations suggest a simplified or highly modified organization of cell polarity and adhesion systems in ctenophores compared to other nonbilaterian animals with expanded diversity of cadherins. Despite this apparent reduction, the *M. leidyi and H. californiensis* cadherin repertoires still include one PCDH. In bilaterians, protocadherins play key roles in nervous system development and neuronal wiring (Vanhalst et al. 2005; Pancho et al. 2020). The identification of a PCDH in neuron-bearing ctenophores, combined with their absence in unicellular organisms, placozoans, and sponges, suggests that PCDHs emerged early in animal evolution, coincident with the appearance of neurons, and were subsequently elaborated and co-opted for neural functions in specific lineages. Surprisingly, PCDHs are absent in arthropods (Hulpiau and van Roy 2011). In these lineages, some functions associated with PCDHs of vertebrates (eg neuronal self-recognition and circuit specificity) are instead largely mediated by alternative adhesion systems, most notably by Down syndrome cell adhesion molecules (Dscam), which generate extensive molecular diversity through alternative splicing (Hattori et al. 2007). This highlights that PCDHs represent one evolutionary solution for neuronal wiring, rather than a universal requirement. Such lineage-specific innovations illustrate how diversification within the cadherin superfamily enabled the progressive elaboration of cell–cell recognition systems. Thus, the expansion and functional specialization of cadherins during early animal evolution may have shaped the transition from cell aggregation to stable, developmentally regulated multicellularity.

## Materials and methods

### Identification and phylogeny of eukaryotic CCC components

To identify homologs of cadherin and catenin proteins across eukaryotic organisms, we trained HMMs using a curated set of eukaryotic proteomes from a previous study (Senatore et al. 2025). For HMM profile construction, cadherin, β-catenin, and α-catenin protein sequences were retrieved from the NCBI database and selected to represent major metazoan lineages, including nonbilaterians, protostomes, and deuterostomes (Sayers et al. 2021). The sequences used to build the HMMs are provided in FASTA format in Data S1 (cadherins), Data S2 (Vin-motif), and Data S3 (ARM domains). Following HMM searches, redundant hits were removed using CD-HIT (Li and Godzik 2006) with a 99.9% sequence identity threshold. The final datasets are provided in Data S4 (cadherins), Data S5 (Vin-motif), and Data S6 (ARM domains). For phylogenetic inference, sequences were aligned using MAFFT v7.490 (Katoh and Standley 2013), trimmed with TrimAl v1.4.1 using the gappyout mode (Capella-Gutiérrez et al. 2009), and maximum-likelihood phylogenies were inferred with IQ-TREE2 v2.2.2.6 (Minh et al. 2020). Node support was assessed using 1,000 replicates of the approximate Bayes test and is reported as percentages (aBayes; Anisimova et al. 2011). Gene trees were visualized and annotated using FigTree v1.4.4, and final figures were assembled in Inkscape. Raw phylogenetic trees in Nexus format are provided in Data S7 (Vin-Motif), Data S8 (ARM domains), and Data S9 (major cadherin families).

### Annotation of the cadherin repertoire in ctenophores

The homologs of CCC components identified in ctenophores from HMMS were validated using BLAST. Cadherin architectures were annotated using InterPro v101.0 (Paysan-Lafosse et al. 2023) and SMART v9.0 (Letunic and Bork 2025). Transmembrane domains were predicted using Phobius v1.01 (Kall et al. 2007). To identify conserved cytoplasmic motifs, MEME analyses were conducted using curated sets of classical cadherins and PCDHs from representative metazoan species, together with the ctenophore cadherin candidates (Bailey et al. 2015). Any protein sequence containing at least two EC repeats was classified as a cadherin, and most of these sequences also possessed a transmembrane domain. Classical cadherin sequences were aligned with MAFFT and visualized using Jalview (Waterhouse et al. 2009; Katoh and Standley 2013). Cadherin-related genes were named according to their gene IDs and are presented in increasing numerical order (Table S1). This ordering is used solely for descriptive purposes and does not imply evolutionary or functional relationships.

### Y2H experiments

The *M. leidyi* Y2H cDNA library was constructed using the Make Your Own “Mate & Plate” Library System (Takara Bio USA, Mountain View, CA, cat. 630490). Total RNA was extracted from a few thousand embryos at various developmental stages using TRIzol (Sigma, cat. T9424) to maximize transcript diversity. The Y2H screens were performed according to the manufacturer's instructions using the coding sequences of the intracellular domain of *Ml*-CDH (amino acids 888 to 1180) and the full length of *Ml*α-catenin, which had been previously cloned into the pGEM-T Easy vector (Promega). These genes were subsequently subcloned into the bait vector pGBKT7, N-terminally tagged to the Gal4 DNA-binding domain using the NEBuilder HiFi DNA Assembly (New England Biolabs, cat. E2621). The Y2H screenings were carried out using the Matchmaker Gold Yeast Two-Hybrid System (Takara Bio USA, Mountain View, CA, cat. 630489), following the manufacturer's protocol. For the directed Y2H screens, the intracellular domain of *Ml*-CDH and the full-length *Ml*α-catenin were cloned into the prey vector pGADT7, N-terminally fused to the Gal4 DNA-activation domain, using the same cloning strategy as described above. These prey constructs were co-transformed with minimal interaction domains, predicted from the preliminary Y2H screens, cloned into the bait vector pGBKT7. To limit the detection of false-positive interactions inherent to the Y2H assay, all interaction combinations were assessed using multiple independent reporter genes. Primer sequences are listed in Table S3.

Quantitative analysis of the β-galactosidase activity for wild-type and mutated CCC components was done in biological triplicate using the yeast β-galactosidase assay kit (Thermo Fisher USA, Rockford, IL, cat. 75768). All primers used are listed in Table S4.

## Supplementary Material

msag123_Supplementary_Data

## Data Availability

The data underlying this article are available in the article and in its online supplementary material.
