# Supplementary material for: Evidence for an early cadherin–catenin interaction network in ctenophores
Source: Mol Biol Evol. 2026 May 22;43(6):msag123. doi: 10.1093/molbev/msag123 (PMC13245172; doi:10.1093/molbev/msag123)
Supplement: msag123_Supplementary_Data [file msag123_supplementary_data.zip › Supplementary data.pdf]

Supplementary data for

Evidence for an Early Cadherin–Catenin Interaction Network in Ctenophores

Lucas J. Guttieres*, Anhadvir Singh, Adriano Senatore, & Mark Q. Martindale

Lucas J. Guttieres
Email: lguttieres@whitney.ufl.edu

This PDF file includes:

Figures S1 to S9
Tables S1 to S4
Supplementary references

Other supporting materials for this manuscript:

Datasets S1 to S9

Dataset S1 (separate file). Provides protein sequences of metazoan cadherins used to generate a profile HMM model.

Dataset S2 (separate file). Provides protein sequences of metazoan Vinculin-motif containing used to generate a profile HMM model.

Dataset S3 (separate file). Provides protein sequences of metazoan β-catenin and p120 used to generate a profile HMM model.

Dataset S4 (separate file). Provides protein sequences of identified cadherins from eukaryotic organisms.

Dataset S5 (separate file). Provides protein sequences of identified Vinculin-motif containing from eukaryotic organisms.

Dataset S6 (separate file). Provides protein sequences of identified ARM-domain containing from eukaryotic organisms.

Dataset S7 (separate file). Provides the raw phylogenetic tree, in nexus format, of Figure 2B.

Dataset S8 (separate file). Provides the raw phylogenetic tree, in nexus format, of Figure 2C.

Dataset S9 (separate file). Provides the raw phylogenetic tree, in nexus format, of Figure 4.

Figures

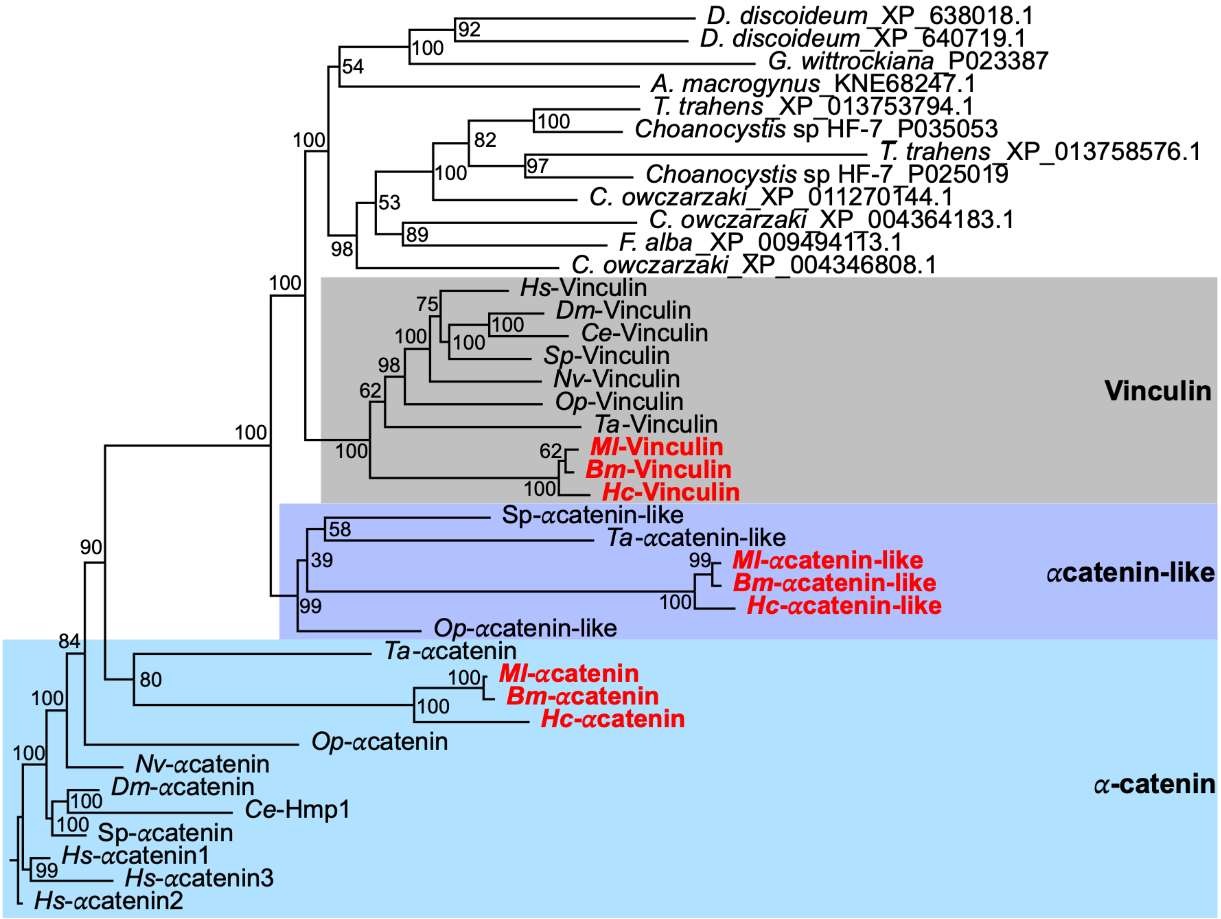

Figure S1. Detailed tree of the selected Vin-motif sequences from Figure 2B. Ctenophore proteins are highlighted in red. Tree and node support values were obtained as explained in the Material and Methods section. Species abbreviations: *Bm*, *Bolinopsis microptera*; Ce, *Caenorhabditis elegans*; *Dm*, *Drosophila melanogaster*; *Hc*, *Hormiphora californiensis*; *Hs*, *Homo sapiens*; *Ml*, *Mnemiopsis leidyi*; *Nv*, *Nematostella vectensis*; *Op*, *Oscarella pearsei*; *Sp*, *Strongylocentrotus purpuratus*; *Ta*, *Trichoplax adhaerens*.

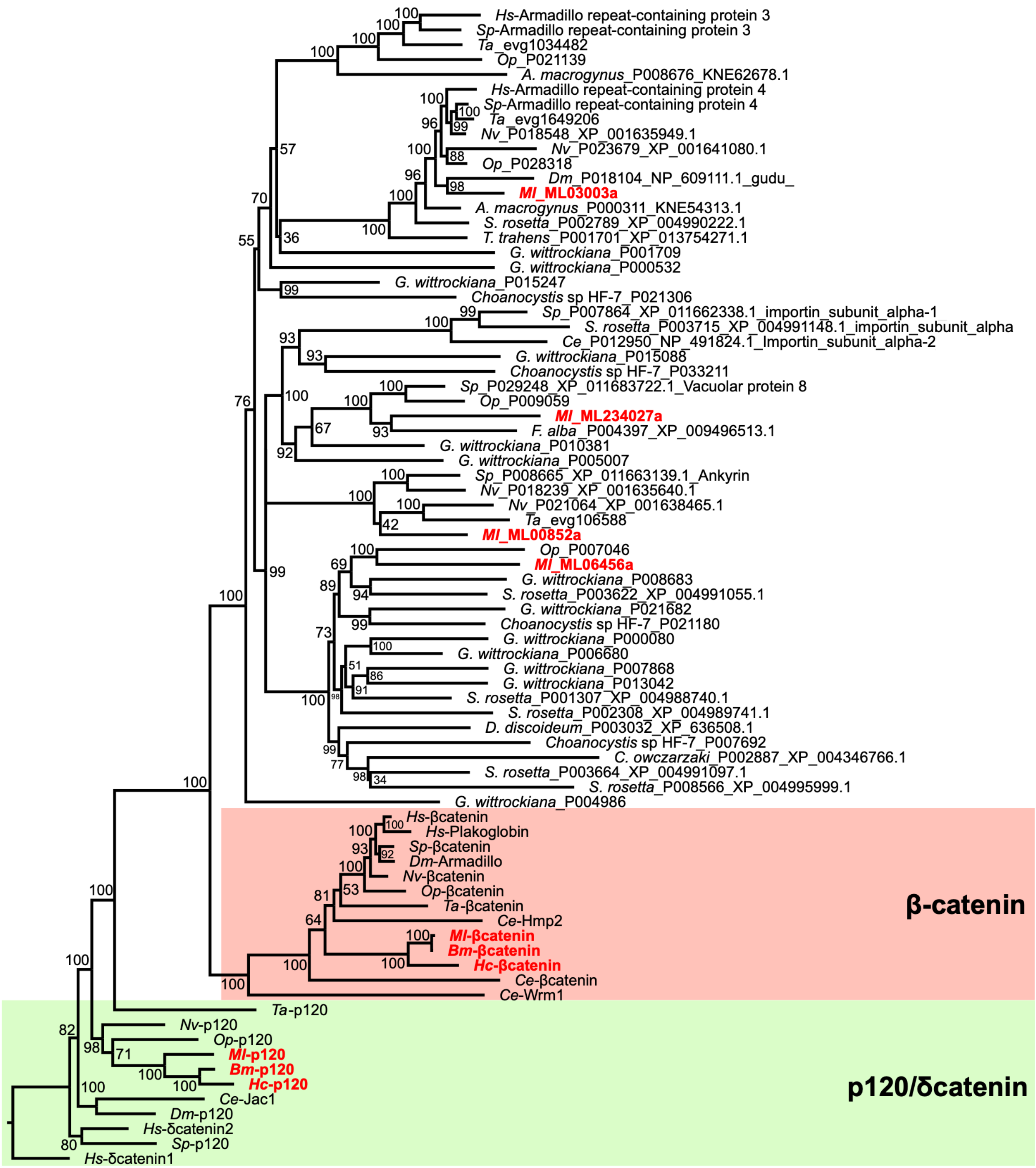

Figure S2. Detailed tree of the selected ARM-domain containing sequences from Figure 2C. Ctenophore proteins are highlighted in red. Tree and node support values were obtained as explained in the Material and Methods section. Species abbreviations are as Fig. S1.

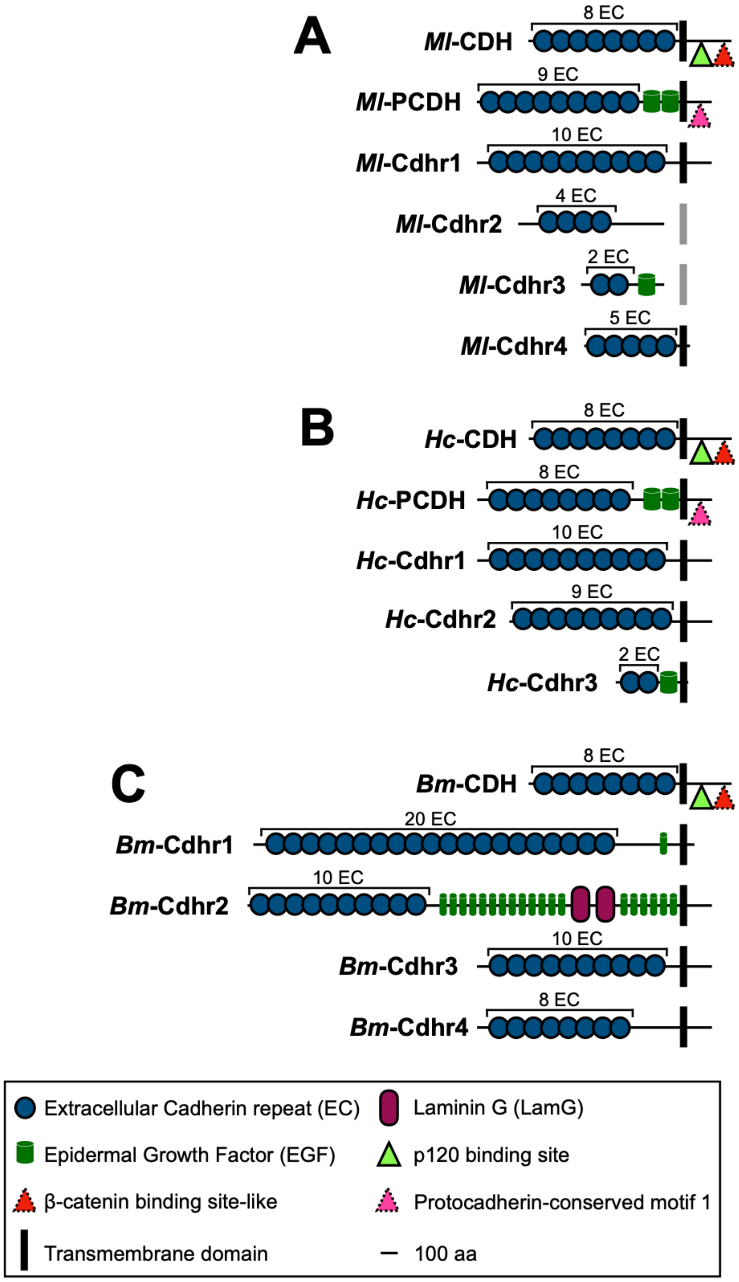

Figure S3. Cadherin repertoire for the three ctenophore species discussed in this study A) *Mnemiopsis leidyi*, B) *Hormiphora californiensis*, and C) *Bolinopsis microptera*. Cadherin architectures were annotated using InterPro v101.0 (Paysan-Lafosse et al. 2022), and SMART v9.0 (Letunic and Bork 2025). Transmembrane domains were predicted using Phobius v1.01 (Kall et al. 2007). Our analysis revealed the presence of

one non-classical cadherin-like protein in the tree ctenophore species, and one protocadherin (PCDH) in *M. leidyi* and *H. californiensis*. The remaining cadherins were defined as cadherin-related (Cdhr) genes, containing EC repeats but lacking the defining features of established cadherin subfamilies. The absence of a predicted transmembrane domain in two Cdhr genes in *M. leidyi* may reflect incomplete genome assembly or gene annotation limitations (Ryan et al. 2013).

Name	Start	*p*-value	Sites
Bf-PCDH2	952	3.09e-10	DDSLNTVLSN HDSGRGDS DREVRANSAC
Bf-PCDH1	1014	3.09e-10	DSGRGSVHSN HDSGRGDS DKEVRSSPTA
Hs-PCDH10	909	5.09e-10	AFQEADIVSS KDSGHGDS EQGDSDHDAT
Hs-PCDH17	888	4.28e-9	TFKDPERASL RDSGHGDS DQADSDQDTN
Ac-PCDH	978	2.03e-8	NSVTSGETTT SDSGRGGS EDDIPLPPIA
Nv-PCDH	937	3.02e-8	SSEGFSSGDE LDSGKGDS SIASSPYTSH
Hs-PCDH9	1064	5.55e-8	HLPDGSQESC SDSGLGDH EPVGSGTLIS
Hc-PCDH	1211	9.46e-8	ILPGDQIRRD KDSGLAGS LKSLCHFDTS
Ml-PCDH	1308	9.46e-8	PNALECQRRD KDSGLAGS LNTLCHFDMD
Sp-PCDH	612	3.79e-7	ILTDAVFRID EDSGRIYS TAELDREIQE

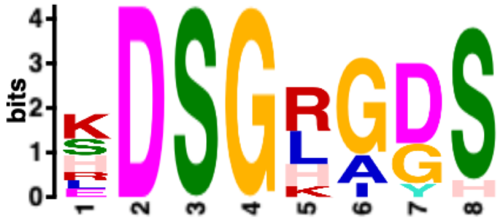

Figure S4. MEME motif analysis of the Conserved protocadherin Motif 1 in selected protocadherins from the same dataset as previously used, in which we added *Ml*-PCDH and *Hc*-PCDH identified in our study (Hulpiau and van Roy 2011; Bailey et al. 2015). Species abbreviations are as Fig. S1 in which we added *Bf*, *Branchiostoma floridae*; *Ac*, *Aplysia californica*.

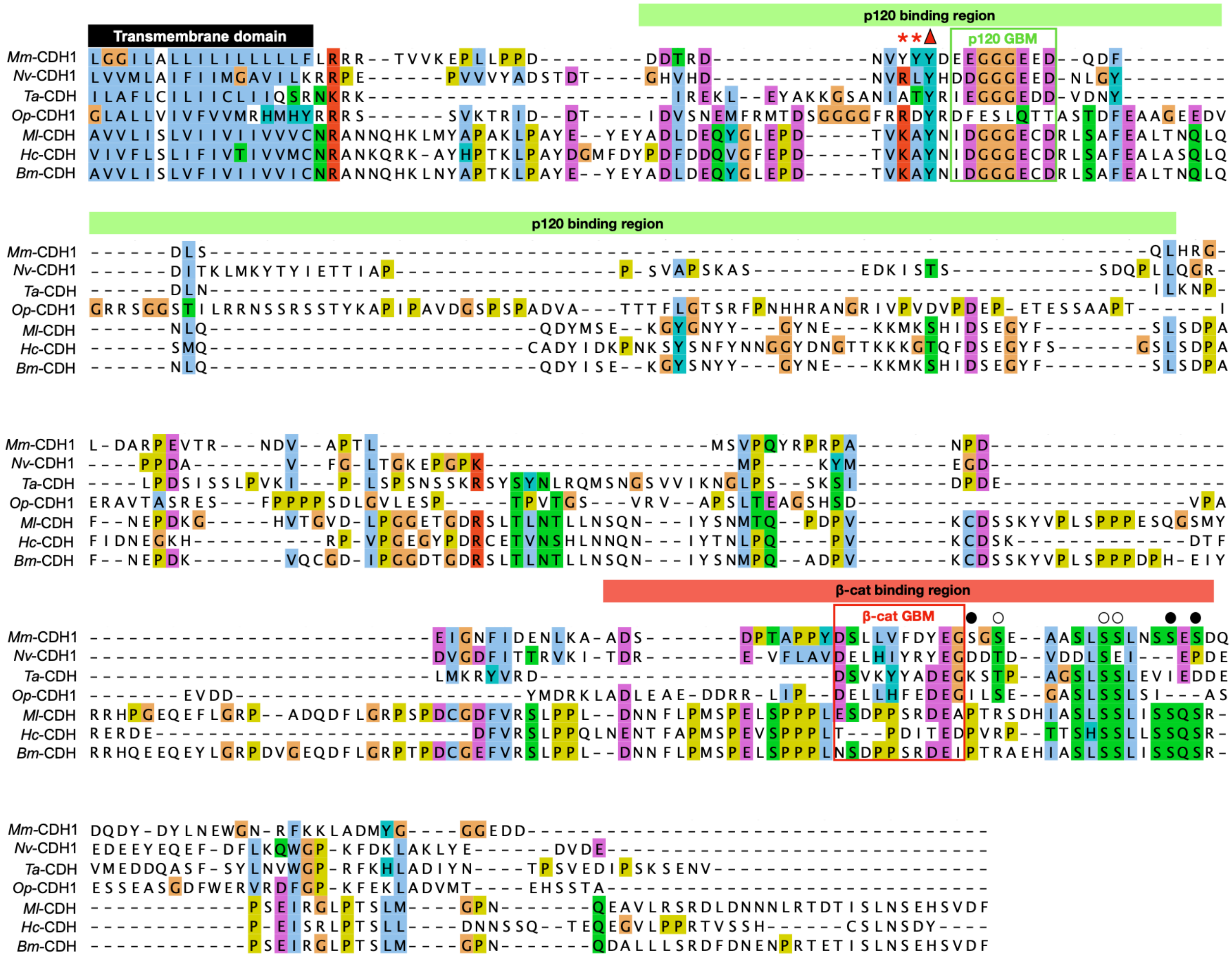

Figure S5. Alignment of the intracellular domain of representative classical cadherins. Following the transmembrane domain (black box), two tyrosine residues that can be phosphorylated to recruit Hakai are indicated by red asterisks (Fujita et al. 2002). A third tyrosine, shown with a red triangle, can be phosphorylated to enhance the stability of the interaction between p120-catenin and the classical cadherin cytoplasmic tail. The p120-catenin binding region, including the Groove Binding Motif (GBM), is highly conserved across metazoans (green box). The β-catenin binding region (red box) is also well conserved, although a few amino acid substitutions are observed within the β-catenin GBM of ctenophore cadherins. Residues phosphorylated by Casein Kinase II (CK2) and Glycogen Synthase Kinase 3β (GSK3β), which regulate cell–cell adhesion,

are indicated by filled and open circles, respectively. Species abbreviations are as Fig. S1 in which we added *Mm*, *Mus musculus*.

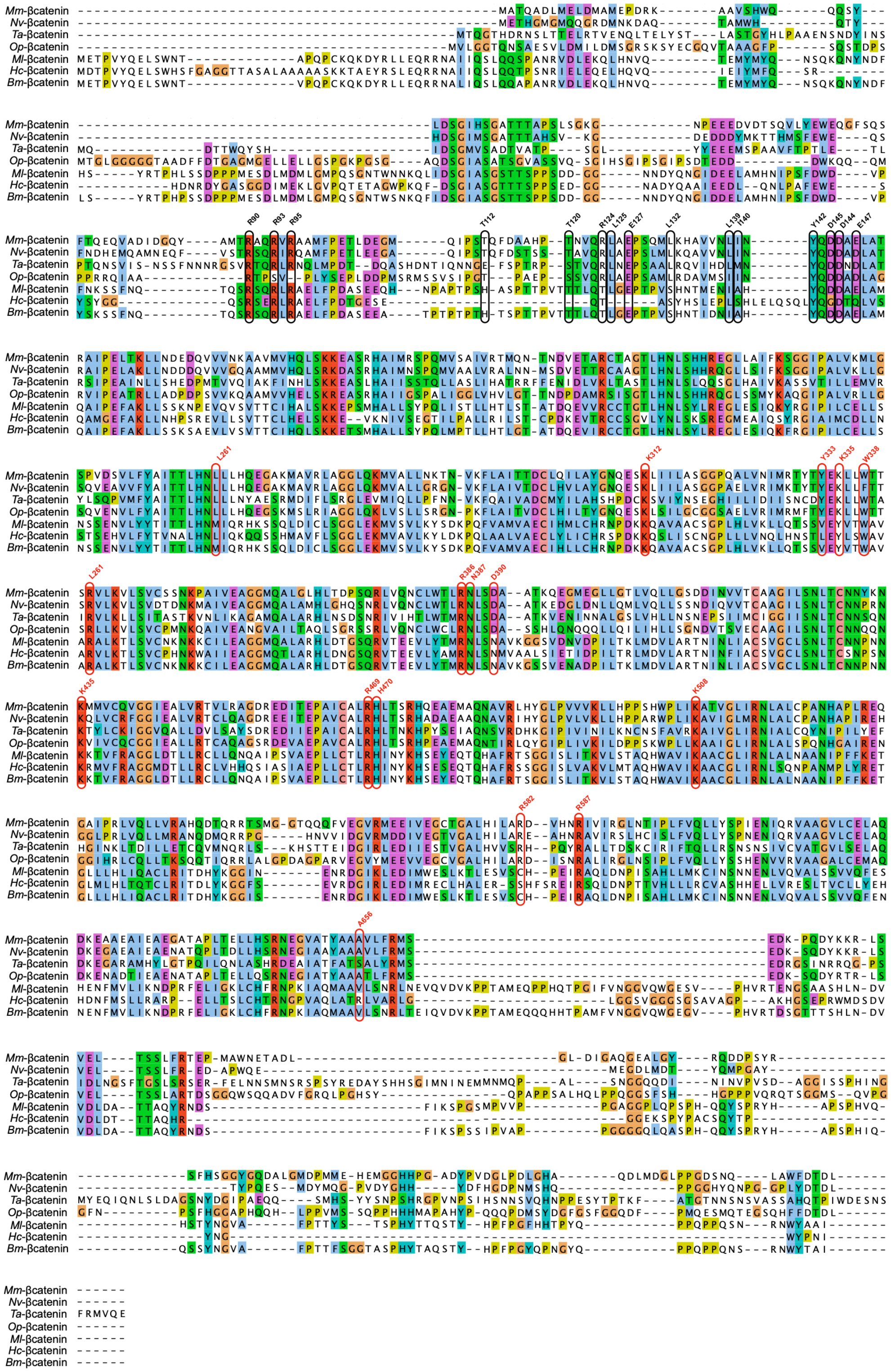

Figure S6. Alignment of selected metazoan β-catenin. Critical residues involved in the interaction between β-catenin and α-catenin are localized in the N-Terminus side and are indicated in black. All residues known to be involved in the interaction between β-catenin and E-cadherin are indicated in red (Belahbib et al. 2018). Species abbreviations are as Fig. S5.

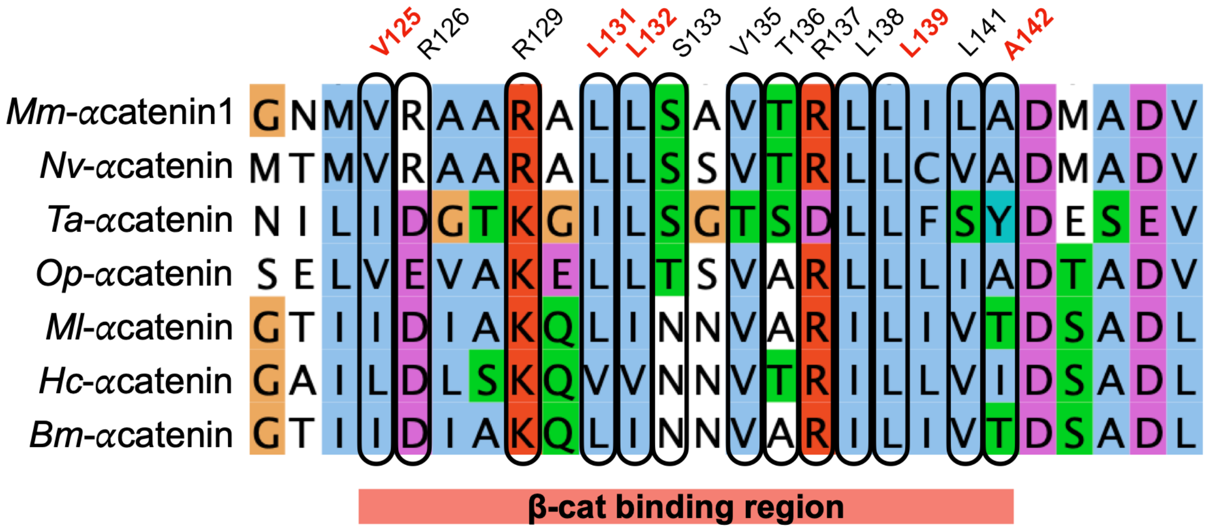

Figure S7. Alignment of the β-catenin–binding region of representative α-catenin from major metazoan groups. The 13 residues known to mediate the interaction between α-catenin and β-catenin are boxed in black. Alanine substitution of the five highlighted residues was previously shown to cause a strong reduction in binding between the two proteins (Huber et al. 1997). Species abbreviations are as Fig. S5.

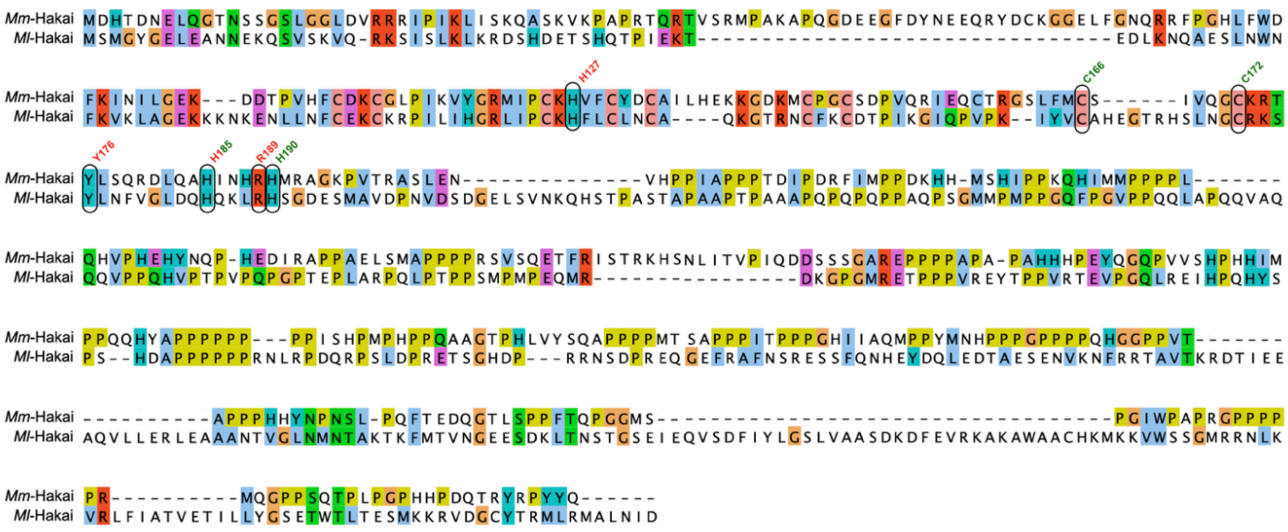

Figure S8. Alignment of mouse and *Mnemiopsis leidyi* Hakai sequences. Residues implicated in binding to epithelial cadherin (H127, Y176, H185, R189) are highlighted in red. Residues forming the Hakai phosphotyrosine-binding (C166, C172, H185, H190) are highlighted in green (Mukherjee et al. 2012). H185 is highlighted in both red and green, as it participates in both cadherin binding and phosphotyrosine recognition.

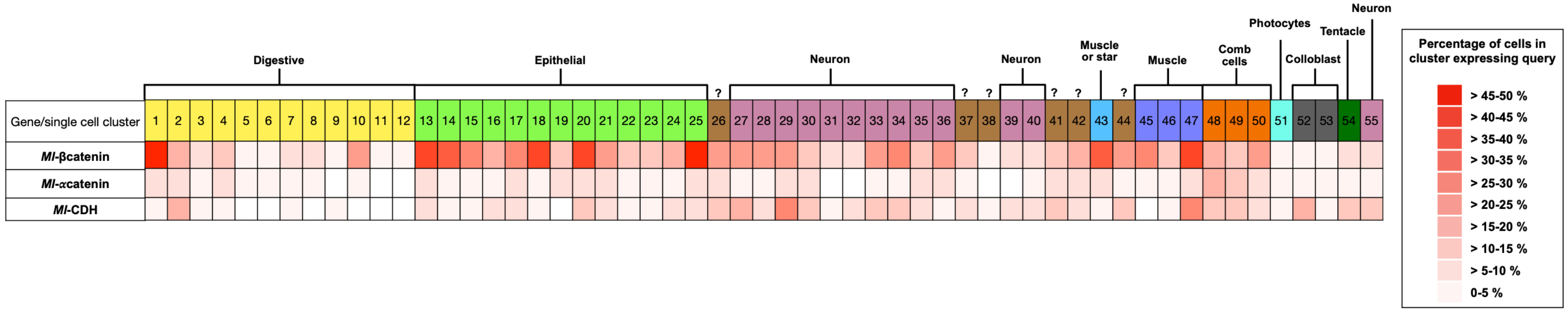

Figure S9. Counts of Cadherin-Catenin complex genes expression in metacells single cell RNA-Seq data, sorted by percentage of cells in a metacell expressing the gene (Sebé-Pedrós et al. 2018).

Tables

Table S1. Gene annotation of ctenophore cadherins. Vinculin-motif–containing, β-catenin, and p120 protein sequences from *Bolinopsis microptera* retrieved from NCBI and discussed in the main text are also included (Sayers et al. 2021).

**Species**	**Gene ID**	**Gene identification**	**Database**
Mnemiopsis leidyi	ML092621a	*Ml*-CDH	https://research.nhgri.nih.gov/mnemiopsis/
Mnemiopsis leidyi	ML00359a	*Ml*-PCDH	https://research.nhgri.nih.gov/mnemiopsis/
Mnemiopsis leidyi	ML07213a	*Ml*-Cdhr1	https://research.nhgri.nih.gov/mnemiopsis/
Mnemiopsis leidyi	ML24141a	*Ml*-Cdhr2	https://research.nhgri.nih.gov/mnemiopsis/
Mnemiopsis leidyi	ML24143a	*Ml*-Cdhr3	https://research.nhgri.nih.gov/mnemiopsis/
Mnemiopsis leidyi	ML106621a	*Ml*-Cdhr4	https://research.nhgri.nih.gov/mnemiopsis/
Hormiphora californiensis	H.cal_Hcv1.av93.c4.g318.i1	*Hc*-CDH	http://ryanlab.whitney.ufl.edu/Hormiphora/
Hormiphora californiensis	H.cal_Hcv1.av93.c6.g1171.i1	*Hc*-PCDH	http://ryanlab.whitney.ufl.edu/Hormiphora/
Hormiphora californiensis	H.cal_Hcv1.av93.c4.g648.i1	*Hc*-Cdhr1	http://ryanlab.whitney.ufl.edu/Hormiphora/
Hormiphora californiensis	H.cal_Hcv1.av93.c7.g900.i1	*Hc*-Cdhr2	http://ryanlab.whitney.ufl.edu/Hormiphora/
Hormiphora californiensis	H.cal_Hcv1.av93.c6.g1172.i1	*Hc*-Cdhr3	http://ryanlab.whitney.ufl.edu/Hormiphora/
Bolinopsis microptera	XP_063677608.1	*Bm*-CDH	https://www.ncbi.nlm.nih.gov
Bolinopsis microptera	XP_063682391.1	*Bm*-Cdhr1	https://www.ncbi.nlm.nih.gov
Bolinopsis microptera	XP_063677767.1	*Bm*-Cdhr2	https://www.ncbi.nlm.nih.gov
Bolinopsis microptera	XP_063678775.1	*Bm*-Cdhr3	https://www.ncbi.nlm.nih.gov
Bolinopsis microptera	XP_063684011.1	*Bm*-Cdhr4	https://www.ncbi.nlm.nih.gov
Bolinopsis microptera	XP_063682934.1	*Bm*-βcatenin	https://www.ncbi.nlm.nih.gov
Bolinopsis microptera	XP_063685569.1	*Bm*-p120	https://www.ncbi.nlm.nih.gov
Bolinopsis microptera	XP_063682306.1	*Bm*-αcatenin	https://www.ncbi.nlm.nih.gov
Bolinopsis microptera	XP_063680290.1	*Bm*-αcatenin-like	https://www.ncbi.nlm.nih.gov
Bolinopsis microptera	XP_063685747.1	*Bm*-Vinculin	https://www.ncbi.nlm.nih.gov

Table S2. Full list of the predicted binding partners from preliminary yeast 2-hybrid screenings using the full-length coding sequence of *Ml*-αcatenin and the intracellular domain of *Ml*-CDH as baits. The number of positive clones and the minimal interacting domains are indicated for all predicted binding partners. Green boxes correspond to interactions confirmed by directed Y2H assays, while red boxes indicate candidates that fail to display direct physical interactions upon re-testing.

Gene ID	Putative gene identification	Colony count	Minimal interacting domain (aa)	Directed Y2H
***Ml*-αcatenin**				
ML073715a	β-catenin	22	134-279	
ML148910a	Vinculin	8	31-205	
ML084414a	Merlin	7	1-118	
ML31032a	Afadin	5	790-1004	
ML02953a	Diaphanous	4	312-462	
ML25062a	α-actinin	4	260-753	
ML26358a	Actin, Alpha skeletal muscle 1	4	1-375	
ML18937a	none	1	40-98	
ML13973a	60S ribosomal protein	1	14-284	
ML35935a	Actin cytoplasmic	1	1-375	
ML23182a	Snurportin-1	1	700-940	
ML234550a	Short transient receptor potential channel 1 TRPC-1	1	450-809	
ML00121a	Myosin-Vb	1	1380-1630	
		**Total:** 60		
**Intracellular domain of *Ml*-CDH**				
ML073715a	β-catenin	12	317-610	
ML009118a	Hakai	4	24-215	
ML13973a	60S ribosomal protein	4	3-178	
ML18937a	none	3	31-116	
ML234550a	Short transient receptor potential channel 1 TRPC-1	3	100-290	
ML002622a	p120	1	244-546	
ML040712a	IQ motif–containing GTPase-activating protein 1 IQGAP-1	1	13-176	
		**Total:** 28		

Table S3. Primers used for amplification of the genes of interest from cDNA, yeast 2-hybrid screens, and directed Y2H experiments. Lowercase letters indicate sequences homologous to the backbone vector for NEBuilder HiFi DNA Assembly. Restriction sites are indicated in bold.

**cDNA amplification**	
pGEM_*Ml*-αcatenin	F: ATGCTTCAAACTTTTTCCTCTATCA<br>R: TTACTTCTTGAAAACTTTTTTCCGTTT
pGEM_ *Ml*-CDH (888-1180)	F: GAATTCAACCAACACAAGCTGATGTACGC<br>R: AAAATCTACCGAATGTTCGGAATTCAACG
**Y2H screens**	
pGBKT7_*Ml*-αcatenin	F: gcatatggccatggaggcc**gaattc**ATGCTTCAAACTTTTTCCTCTATCATAGACTTTGA<br>R: gccgctgcaggtcgaa**ggatcc**CTTCTTGAAAACTTTTTTCCGTTTGCTGC
pGBKT7_ *Ml*-CDH (888-1180)	F: gcatatggccatggaggcc**gaattc**AACCAACACAAGCTGATGTACG<br>R: gccgctgcaggtcgac**ggatcc**AAAATCTACCGAATGTTCGGAATTCAACG
**Directed Y2H**	
***Ml*-αcatenin**	
pGADT7_*Ml*-αcatenin	F: atggccatggaggccagt**gaattc**ATGCTTCAAACTTTTTCCTCTATCATAGACTTTGA<br>R: tctgcagctcgagctcgat**ggatcc**CTTCTTGAAAACTTTTTTCCGTTTGCTGC
pGBKT7_*Ml*-βcatenin (134-279)	F: gcatatggccatggaggcc**gaattc**AACGGGATTGACGAAGAGC<br>R: gccgctgcaggtcgaa**ggatcc**ATGGAGTAAGGTAGATATAAGTTGA
pGBKT7_*Ml*-Vinculin (31-205)	F: gcatatggccatggaggcc**gaattc**CGATGCCGTCATGGCTGACTG<br>R: gccgctgcaggtcgaa**ggatcc**CTCGAGCTCAAGACGTCCAGC
pGBKT7_*Ml*-Merlin (1-118)	F: gcatatggccatggaggcc**gaattc**ATGGCTCATAGTTTTACTGGATC<br>R: gccgctgcaggtcgaa**ggatcc**TGTTATGTCCTGGATCAGGTCGT
pGBKT7_*Ml*-Afadin (790-1004)	F: gcatatggccatggaggcc**gaattc**AGGCCTCATTCTAGGGATGG<br>R: gccgctgcaggtcgaa**ggatcc**CAGGGATAGGTAGTTTGAGAGGAGG
pGBKT7_*Ml*-Diaphanous (312-462)	F: gcatatggccatggaggcc**gaattc**GAACTCACGCAAAAGCTGGGA<br>R: gccgctgcaggtcgaa**ggatcc**AATGAGTCGCTTTTTTTCTTCGTCA
pGBKT7_*Ml*-αactinin (260-753)	F: gcatatggccatggaggcc**gaattc**TGTAAAGTCCTCAACGTCAACCAGG<br>R: gccgctgcaggtcgaa**ggatcc**CTTGTCGAAATGATCGAAGGC
pGBKT7_*Ml*-Actin, alpha skeletal muscle	F: gcatatggccatggaggcc**gaattc**ATGGATGATGATGTAGCTGCCC<br>R: gccgctgcaggtcgaa**ggatcc**GAAGCATTTCCTGTGGACAATGC
pGBKT7_ ML18937a (40-98)	F: gcatatggccatggaggcc**gaattc**CACTATGAGCTTTTAGACCCGGC<br>R: gccgctgcaggtcgaa**ggatcc**ATTTACAGTAGCCCCATCCTCAATC
pGBKT7_*Ml*-60S ribosomal protein (14-284)	F: gcatatggccatggaggcc**gaattc**AAGAGATATCAGGTCAAGTATCGAC<br>R: gccgctgcaggtcgaa**ggatcc**TTGTTGGGCAACTCTTCCCTTG
pGBKT7_*Ml*-Actin cytoplasmic	F: gcatatggccatggaggcc**gaattc**ATGGATGAGGAAGACACTCCCG<br>R: gccgctgcaggtcgaa**ggatcc**GAAGCACTTTCTGTGAACGATGG
pGBKT7_*Ml*-Snurportin 1 (740-940)	F: gcatatggccatggaggcc**gaattc**AAGTCCTACTACAGTGACGCTC<br>R: gccgctgcaggtcgaa**ggatcc**AGGAAATTCCTCAAATGAACCTG
pGBKT7_*Ml*-TRPC 1 (450-809)	F: gcatatggccatggaggcc**gaattc**GAATACATCGACTTCATGGGAACTC<br>R: gccgctgcaggtcgaa**ggatcc**CTTGCCGACGGCCATGTT

pGBKT7_*Ml*-Myosin Vb (1380-1665)	F: gcatatggccatggaggcc**gaattc**GCCCTGAAGAACTACGATCTGTC<br>R: gccgctgcaggtcgaa**ggatcc**CGGAAGGGTTACCATGGTAAAGTC
**Intracellular domain of *Ml*-CDH**	
pGADT7_ *Ml*-CDH (888-1180)	F: atggccatggaggccagt**gaattc**AACAACCAACACAAGCTGATGTACG<br>R: tctgcagctcgagctcgat**ggatcc**AAAATCTACCGAATGTTCGGAATTCAACG
pGBKT7_*Ml*-βcatenin (317-610)	F: gcatatggccatggaggcc**gaattc**CCGATACTATGTGAGCTGCTGAG<br>R: gccgctgcaggtcgaa**ggatcc**TTTGAAGAAGGGAATGTTGGCAGC
pGBKT7_ *Ml*-Hakai (24-215)	F: gcatatggccatggaggcc**gaattc**TCCATATCTTTGAAGCTGAAACG<br>R: gccgctgcaggtcgaa**ggatcc**CATCATTCCAGATGGCTGTGC
pGBKT7_ *Ml*-60S ribosomal protein (3-178)	F: gcatatggccatggaggcc**gaattc**TTCGTCAAGGTTGTCAAGAAC<br>R: gccgctgcaggtcgaa**ggatcc**TCTCTTGGAGGAGTGGGGG
pGBKT7_ ML18937a (31-116)	F: gcatatggccatggaggcc**gaattc**TCTTGGTCTGTTGAGGAAGACTC<br>R: gccgctgcaggtcgaa**ggatcc**GCTGATATTTGTTTCTACGAGGAC
pGBKT7_ *Ml*-p120 (244-546)	F: gcatatggccatggaggcc**gaattc**CAGATCTCTCACAACAACTCCCGA<br>R: gccgctgcaggtcgaa**ggatcc**CTTCCCCTCCTCGGGAGT
pGBKT7_ *Ml*-TRPC1 (100-290)	F: gcatatggccatggaggcc**gaattc**GGACACGAAACTCTCCGAATAG<br>R: gccgctgcaggtcgaa**ggatcc**GTTGACCCAATCGTTGCC
pGBKT7_ *Ml*-IQGAP1 (13-176)	F: gcatatggccatggaggcc**gaattc**CAAGAAAGAAAAGATTACTTGAACA<br>R: gccgctgcaggtcgaa**ggatcc**GTTCTGCGGCATAGTAAACAGT

Table S4. Primers used for site-directed mutagenesis and truncated α-catenin. Nucleotides that were altered to introduce the mutations are highlighted in red. Lowercase letters indicate sequences homologous to the backbone vector pGADT7 for NEBuilder HiFi DNA Assembly. Restriction sites are indicated in lowercase in bold.

Ml-βcatenin (Y221E)	F: CATCCTG**TTC**ATGAGCGATGTTCTCCATGG<br>R: CTCAT**GAA**CAGGATGACGCTGAACTGGC
Ml-βcatenin (K392A)	F: CAGCTTG**AGC**CTTGTCCGGGTTACGATGACAGAGC<br>R: ACAAG**GCT**CAAGCTGTAGCTGCCTGTTCC
Ml-βcatenin (K517A)	F: CGGTCTT**AGC**GTTGTTCGGGTTATTGCAAGTC<br>R: ACAAC**GCT**AAGACCGTATTCCGAGCG
pGADT7_*Ml*-αcatenin (128-777)	F: atggccatggaggccagt**gaattc**GACTCAGCAGATTTGATTCTTA<br>R: tctgcagctcgagctcgat**ggatcc**CTTCTTGAAAACTTTTTTCCGTTTG

Supplementary references:

Bailey TL, Johnson J, Grant CE, Noble WS. 2015. The MEME suite. *Nucleic Acids Res.* 43:W39–W49.

Belahbib H, Renard E, Santini S, Jourda C, Claverie J-M, Borchiellini C, Le Bivic A. 2018. New genomic data and analyses challenge the traditional vision of animal epithelium evolution. *BMC Genomics* 19:393.

Fujita Y, Krause G, Scheffner M, Zechner D, Leddy HEM, Behrens J, Sommer T, Birchmeier W. 2002. Hakai, a c-Cbl-like protein, ubiquitinates and induces endocytosis of the E-cadherin complex. *Nat Cell Biol* 4:222–231.

Huber O, Krohn M, Kemler R. 1997. A specific domain in α-catenin mediates binding to β-catenin or plakoglobin. *J Cell Sci.* 110:1759–1765.

Hulpiau P, Van Roy F. 2011. New insights into the evolution of metazoan cadherins. *Mol Biol Evol.* 28:647–657.

Kall L, Krogh A, Sonnhammer ELL. 2007. Advantages of combined transmembrane topology and signal peptide prediction—the Phobius web server. *Nucleic Acids Res.* 35:W429–W432.

Letunic I, Bork P. 2025. SMART v10: three decades of the protein domain annotation resource. *Nucleic Acids Res.* gkaf1023.

Mukherjee M, Chow SY, Yusoff P, Seetharaman J, Ng C, Sinniah S, Koh XW, Asgar NFM, Li D, Yim D, et al. 2012. Structure of a novel phosphotyrosine-binding domain in Hakai that targets E-cadherin. *The EMBO Journal* 31:1308–1319.

Paysan-Lafosse T, Blum M, Chuguransky S, Grego T, Pinto BL, Salazar GA, Bileschi ML, Bork P, Bridge A, Colwell L, et al. 2023. InterPro in 2022. *Nucleic Acids Res.* 51:D418–D427.

Ryan JF, Pang K, Schnitzler CE, Nguyen A-D, Moreland RT, Simmons DK, Koch BJ, Francis WR, Havlak P, NISC Comparative Sequencing Program, et al. 2013. The genome of the ctenophore *Mnemiopsis leidyi* and its implications for cell type evolution. *Science* 342:1242592.

Sayers EW, Beck J, Bolton EE, Bourexis D, Brister JR, Canese K, Comeau DC, Funk K, Kim S, Klimke W, et al. 2021. Database resources of the National Center for Biotechnology Information. *Nucleic Acids Res.* 49:D10–D17.

Sebé-Pedrós A, Chomsky E, Pang K, Lara-Astiaso D, Gaiti F, Mukamel Z, Amit I, Hejnol A, Degnan BM, Tanay A. 2018. Early metazoan cell type diversity and the evolution of multicellular gene regulation. *Nat Ecol Evol.* 2:1176–1188.
